# Physical Activity and Exercise Patterns After Spontaneous Coronary Artery Dissection: Insights From a Large Multinational Registry

**DOI:** 10.3389/fcvm.2021.642739

**Published:** 2021-06-15

**Authors:** Audry S. Chacin-Suarez, Amanda R. Bonikowske, Jose R. Medina-Inojosa, Rajiv Gulati, Patricia J. Best, Sharonne N. Hayes, Marysia S. Tweet

**Affiliations:** Department of Cardiovascular Medicine, Mayo Clinic, Rochester, MN, United States

**Keywords:** spontaneous coronary artery dissection, exercise prescription, young adult, women cardiovascular disease, physical exertion, exercise

## Abstract

**Objective:** The objective of the study was to assess the physical activity (PA) and exercise patterns among participants in a large multinational spontaneous coronary artery dissection (SCAD) registry. Patients and Methods: Participants with SCAD enrolled from March 2011 to November 2019 completed surveys including details regarding PA and exercise habits prior to SCAD, and PA counseling received from their provider after SCAD. Demographics and clinical characteristics were collected by electronic record review. Exercise prescribed to patients after SCAD was categorized according to exercise components: type, intensity, frequency, time/session, and extreme environmental conditions.

**Results:** We included 950 participants; mean ± age was 46.8 ± 9.5 years old at the time of first SCAD; most (96.3%) were women and (77.0%) attended ≥1 cardiac rehabilitation session. Hyperlipidemia (34.3%), hypertension (32.8%), and elevated body weight (overweight = 27.0%; obesity = 20.0%) were the most common comorbidities. Prior to SCAD, 48.5% performed aerobic exercise ≥3 times/week, and only 32.0% performed strength-building exercise regularly. PA counseling details after SCAD in 299/950 participants showed that most (93.3%) patients received some form of counseling including exercise prescription (EXP), non-specific recommendations, and discouraged from any exercise. Limits regarding exercise type and intensity were the most common advice among participants who received EXP.

**Conclusion:** Insights from our study suggest that only 48% of the patients performed some aerobic exercise three or more times per week, and 32.0% performed strength-building exercises, which suggest that most of them may not be as active as assumed. Furthermore, 70% of the SCAD patients have ≥1 cardiovascular risk factors. We suggest guiding patients based on individual assessment, taking into consideration baseline PA habits, treatment, and risk factors. SCAD-tailored PA guidelines are needed for optimal EXP without compromising patient safety.

## Introduction

Spontaneous coronary artery dissection (SCAD) is a significant cause of myocardial infarction without atherosclerotic coronary artery disease in otherwise healthy and presumably fit young women (1–3). The prevalence of SCAD has been reported in 0.07–0.2% of all coronary angiograms performed (4); however, population-based incidence and prevalence of SCAD remains uncertain (5, 6).

Physical activity (PA) and exercise after cardiovascular events improves aerobic capacity (7), body composition, and overall mental health (8). Supported by a plethora of evidence, cardiac rehabilitation (CR) is a class I guideline-directed intervention that increases quality of life, reduces morbidity and mortality, and decreases recurrent cardiovascular events after acute myocardial infarction (9–12). The relevant studies were primarily of those who had acute myocardial infarction due to atherosclerosis and did not differentiate those individuals from SCAD patients. SCAD pathophysiology represents a heterogeneous clinical entity that might require variations in short- and long-term management strategies (13).

The role of PA and exercise in patients after SCAD is not well-established. Most patients have been advised against performing strenuous physical exertion and prolonged high-intensity or heavy weightlifting activities (14–18); however, there is little evidence to support these recommendations. In the absence of evidence, concerns regarding benefit or harm of PA as it relates to SCAD remain, particularly because patients are at risk of recurrent events (6, 19).

Reports have suggested that early and long-term survival after SCAD is favorable (6, 19, 20). SCAD survivors are thought to be young and highly active (9); therefore, pre-SCAD PA level might play an important role in establishing individualized management and recovery goals. The present study involves a series of patients who were enrolled in a large multinational registry (21). We aimed to (1) assess self-reported PA and exercise habits in patients prior and after SCAD, (2) understand self-reported patterns of PA and exercise prescribed to patients after SCAD, and (3) assess self-reported exercise prescription (EXP) components delivered to patients after SCAD.

## Materials and Methods

### Design and Procedures

We reviewed 998 patients enrolled in the Mayo Clinic SCAD Registry from March 2011 to November 2019 (Supplementary Figure 1). The ongoing SCAD Registry was approved by the Mayo Clinic Institutional Review Board. Participants were enrolled after confirmation of the SCAD diagnosis of coronary angiography and medical record review. Written consent was then obtained from all participants. Participants include patients diagnosed with SCAD at the Mayo Clinic, as well as clinical and self-referrals to the registry. Participants are able to enroll into the registry without a medical visit to the Mayo Clinic, which allows recruitment of patients with SCAD from throughout the United States and the world.

Baseline characteristics of participants were collected by trained nurse abstractors based on electronic record review and initial surveys that included demographics, socioeconomics, clinical characteristics, details regarding unusual PA, and specific questions regarding exercise habits prior to SCAD (Supplementary Figure 2). Unusual PA was defined as any activity that leads to exhaustion, endurance training, elite competitive sports, or vigorous exertion, including extremes of ambient temperature. A follow-up survey was completed by some participants. In this survey, PA habits after SCAD were obtained; open-ended questions regarding PA counseling received from their health care provider at the first encounter after SCAD and subsequent medical encounter were also assessed (Supplementary Figure 3).

### Statistical Analyses

Statistical analyses were performed using JMP statistical software, version Pro 14.1.0 (SAS, Cary, North Carolina). Continuous variables are presented as mean ± SD. Categorical variables are presented as numbers and percentages. The number of responses for each question varied due to incomplete responses (e.g., respondent did not answer a question); for descriptive analyses, percentages were computed with the denominator being the number of responses for a specific item.

From the first survey, close-ended questions (Supplementary Figure 2) assessing exercise habits prior to SCAD were categorized: aerobic exercise and strength-building exercise. Furthermore, aerobic exercise was grouped in categories according to frequency (i.e., none, <3 times per week, ≥3 times per week, or not sure) and time per sessions (i.e., <30 min per session, 31–60 min per session, ≥60 min per session, or not sure) (Supplementary Figure 4).

Qualitative data generated through open-ended questions were analyzed by two investigators (AC-Z and JM-I). These questions from the second survey (Supplementary Figure 3) were analyzed with open coding and categorization of concepts. Regarding PA habits after SCAD, data were grouped into previously described categories (Supplementary Figure 5). “Did not specify” was used in cases where the participant did not provide information about the inquiry.

Supplementary Figure 6 shows the theoretical framework for the open coding during the qualitative analysis of data regarding first and subsequent encounters. Data were categorized into emerging patterns, which were later grouped. Three groups emerged: participants who received PA counseling, participants who did not receive PA counseling, and a third category called “Miscellaneous.” (i.e., participant's answer was ambiguous or not related to the question). Sub-analysis of the group that received PA counseling included three subcategories: formal exercise prescription (EXP), non-specific recommendations, or discouraged from any exercise.

Additionally, the EXP category was sub-analyzed and grouped according to exercise components: type, intensity, frequency, and duration of exercise sessions (time/session) based on definitions of the American College of Sports Medicine guidelines (22). “Extreme environmental conditions” related to exercise performance and “CR exercise program” were added as components of EXP since they were highly reported among participants (Supplementary Figure 6). “CR exercise program” category reflects participants who reported following an exercise plan delivered by a CR program and did not provide specific details regarding exercise components. Exercise components were later categorized into emerging patterns, which were later grouped (Supplementary Figure 6).

## Results

For the purposes of this study, 950 of 998 participants who completed initial surveys were included (Supplementary Figure 1). Overall mean time between the first SCAD event and completion of the surveys was 2.5 ± 3.7 years. A follow-up survey was completed by 299/950 participants; the overall mean time between the first and second survey completion was 2.7 ± 0.9 years. Baseline characteristics of patients with SCAD are shown in Table 1. The mean ± SD age of the participants was 46.8 ± 9.5 years at the time of SCAD and 49.0 ± 10.1 years at the time of the initial survey. Most (*n* = 915, 96.3%) were women, white, not of Hispanic origin (*n* = 877, 92.3%). Geographic distribution of patients is shown in Supplementary Figure 7. The majority (*n* = 572; 60.2%) of the participants resided in the United States. Most (*n* = 717, 77.0%) attended ≥1 CR sessions, and overall average CR attendance was 18.6 ± 11.7 sessions. Hyperlipidemia (*n* = 311, 34.3%), hypertension (*n* = 304, 32.8%), and elevated body weight (overweight = 234, 27.0%; obesity = 173, 20.0%) were the most common cardiovascular risk factors, and 70.1% participants had one or more factors. Less than 2% (*n* = 16) reported current smoking (Table 1).

**Table 1:** Characteristics of patients with spontaneous coronary artery dissection.

**Total population (*n* = 950)**	**Number (%)**
Age (years) at time of SCAD, mean ± SD	46.8 ± 9.5
Age (years) at time of survey, mean ± SD	49.0 ± 10.1
Female	915 (96.3)
**Race—Ethnicity**	
White-non-Hispanic	877 (92.3)
White-Hispanic	24 (2.55)
Asian	12 (1.27)
African American	10 (1.06)
American Indian/Alaskan Native	6 (0.64)
Other	13 (1.38)
Married	773 (82.2)
Yearly income ≥ $50,000	157 (17.1)
Yearly income ≥ $80,000	654 (71.4)
Employed full time	488 (51.9)
Employed part time	182 (19.4)
Completed 12 years of school	924 (99.1)
Completed 16 years of school	657 (70.5)
CR attendance	717 (77.0)
CR sessions, mean ± SD	18.6 ± 11.7
More than 1 SCAD events	144 (15.5)
Hypertension	304 (32.8)
Diabetes mellitus	27 (2.9)
Hyperlipidemia	311 (34.3)
Overweight (BMI = 25.0–29.9)	234 (27.0)
Obesity (BMI ≥ 30.0)	173 (20.0)
BMI, mean ± SD	25.9 ± 5.9
Fibromuscular dysplasia	330 (35.1)
Connective tissue disorder	37 (4.0)
Postmenopausal hormone therapy	173 (18.9)
Fertility treatment	115 (12.7)
Current smoker^a^	16 (1.7)
Former smoker^a^	254 (27.5)
**Exercise habits prior SCAD**	
Aerobic exercise	
None	158 (16.6)
<3 times per week	308 (32.4)
3 or more times per week	461 (48.5)
<30 min/session	81 (17.6)
31–60 min/session	288 (62.5)
>60 min/session	91 (19.7)
Not sure	23 (2.4)
**Strength building exercise**	
No	634 (66.9)
Yes	303 (32.0)
Not sure	11 (1.2)
**Unusual physical activity prior to SCAD**	
No	645 (68.3)
Yes	266 (28.2)
Not sure	33 (3.4)
**Coronary artery affected**	
Left main, left anterior descending artery, or multivessel^b^	584 (63.1)
Left circumflex artery or right coronary artery	206 (22.2)
**Intervention**	
Medical therapy only	533 (57.4)
PCI or CABG	364 (39.4)
**Symptoms and concerns after SCAD**	
Recurrent symptoms of chest pain	495 (53.0)
Chest discomfort or shortness of breath following physical activity	537 (56.8)
Concerned about recurrence of SCAD event	418 (44.8)
Concerned about sudden cardiac death	300 (32.2)

*BMI, body mass index (calculated as weight in kilograms divided by height in meters squared); CABG, coronary artery bypass grafting; PCI, percutaneous coronary intervention; SCAD, spontaneous coronary artery dissection; SD, standard deviation*.

a *Smoking status at the time of SCAD*.

b *High-risk anatomy*.

The left main artery, left anterior descending artery, or multiple vessels were most frequently affected (*n* = 584, 63.1%), and half (*n* = 533, 57.4%) of participants were initially managed with medical therapy only. Over 50% (*n* = 495, 53.0%) recalled having recurrent symptoms of chest pain after SCAD, or chest discomfort/ shortness of breath (*n* = 537, 56.8%) following PA. Furthermore, concerns about SCAD recurrence (*n* = 418, 44.8%) or sudden cardiac death (*n* = 300, 32. 2%) in the future were also reported (Table 1).

### Physical Activity and Exercise Habits Prior and After Spontaneous Coronary Artery Dissection

Table 1 shows exercise habits in patients prior to SCAD. Less than half (*n* = 461, 48.5%) performed aerobic exercise ≥3 times per week, of which 62.5% (*n* = 288) of the participants exercised between 31 and 60 min. Only 19.7% (*n* = 91) reported aerobic exercise sessions longer than 60 min. Of the overall group, the majority (*n* = 634, 66.9%) were not performing strength-building exercise, and most (*n* = 645, 68.3%) participants did not engage in unusual PA prior to SCAD (Table 1).

PA habits after SCAD in 299 participants are shown in Table 2. Half of the participants (*n* = 153, 51.2%) were performing aerobic activities ≥3 times per week. Of this group, most (*n* = 109, 71.2%) participants reported sessions of 31–60 min. Fifty-nine participants (17.4%) were not physically active at all.

**Table 2:** Physical activity habits after SCAD^a^.

**Overall = 299**	
	**Number (%)**
**Aerobic**	
None	52 (17.4)
<3 times per week	11 (3.7)
3 or more times per week	153 (51.2)
<30 min/session	10 (6.5)
31–60 min/session	109 (71.2)
>60 min/session	1 (0.7)
Did not specify frequency	83 (27.7)
**Strength building**	
No	58 (19.4)
Yes	59 (19.7)
Did not specify	182 (60.9)

*SCAD, spontaneous coronary artery dissection*.

a *Qualitative data from SCAD follow-up survey*.

**Figure 1:**
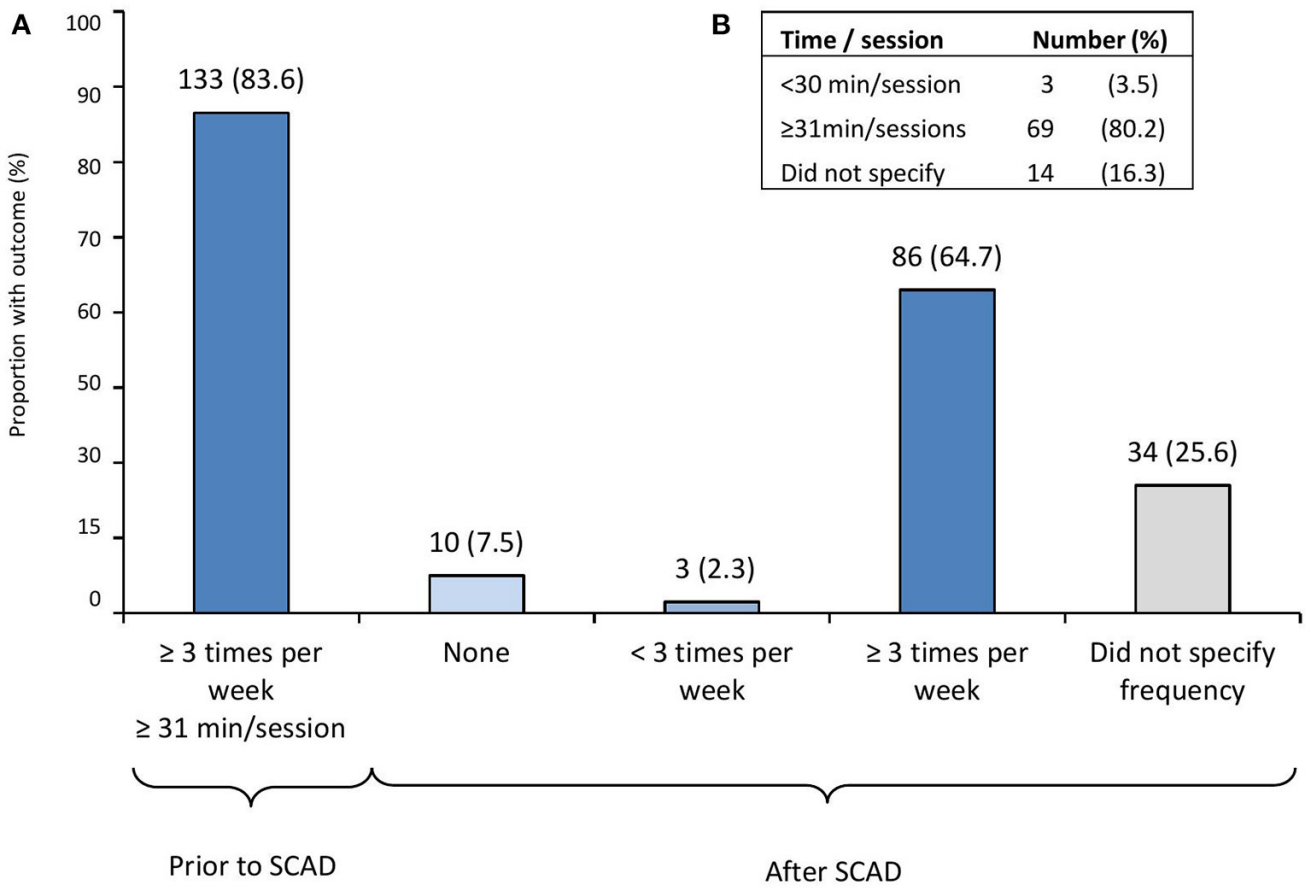
Physical activity and exercise habit changes after SCAD in previously active participants. **(A)** Changes in physical activity and exercise habits among participants performing aerobic exercise ≥3 times per week, with each time/session ≥31 min prior to SCAD (i.e., active participants, *n* = 133). **(B)** Duration of the exercise sessions in participants that remained active ≥3 times per week (*n* = 86; 64.7%) after SCAD. SCAD, spontaneous coronary artery dissection. From 299 participants who completed initial and follow-up surveys, a sub-analysis of changes in physical activity and exercise habits was made among active participants defined as those performing aerobic exercise three or more times per week, with each exercise time/sessions ≥31 min prior to SCAD.

Of the overall 299 participants, a sub-analysis of changes in PA and exercise habits was made (Figure 1A). Among the participants performing aerobic exercise ≥3 times per week, with each time/sessions, ≥31 min prior to SCAD (i.e., aerobically active participants, *n* = 133), <10% (*n* = 10) were not exercising after SCAD, and two-thirds (*n* = 86, 64.7%) remained active ≥3 times per week after SCAD. The duration of the exercise sessions in active participants after SCAD is shown in Figure 1B.

### Physical Activity Counseling and Exercise Prescription After Spontaneous Coronary Artery Dissection

**Figure 2:**
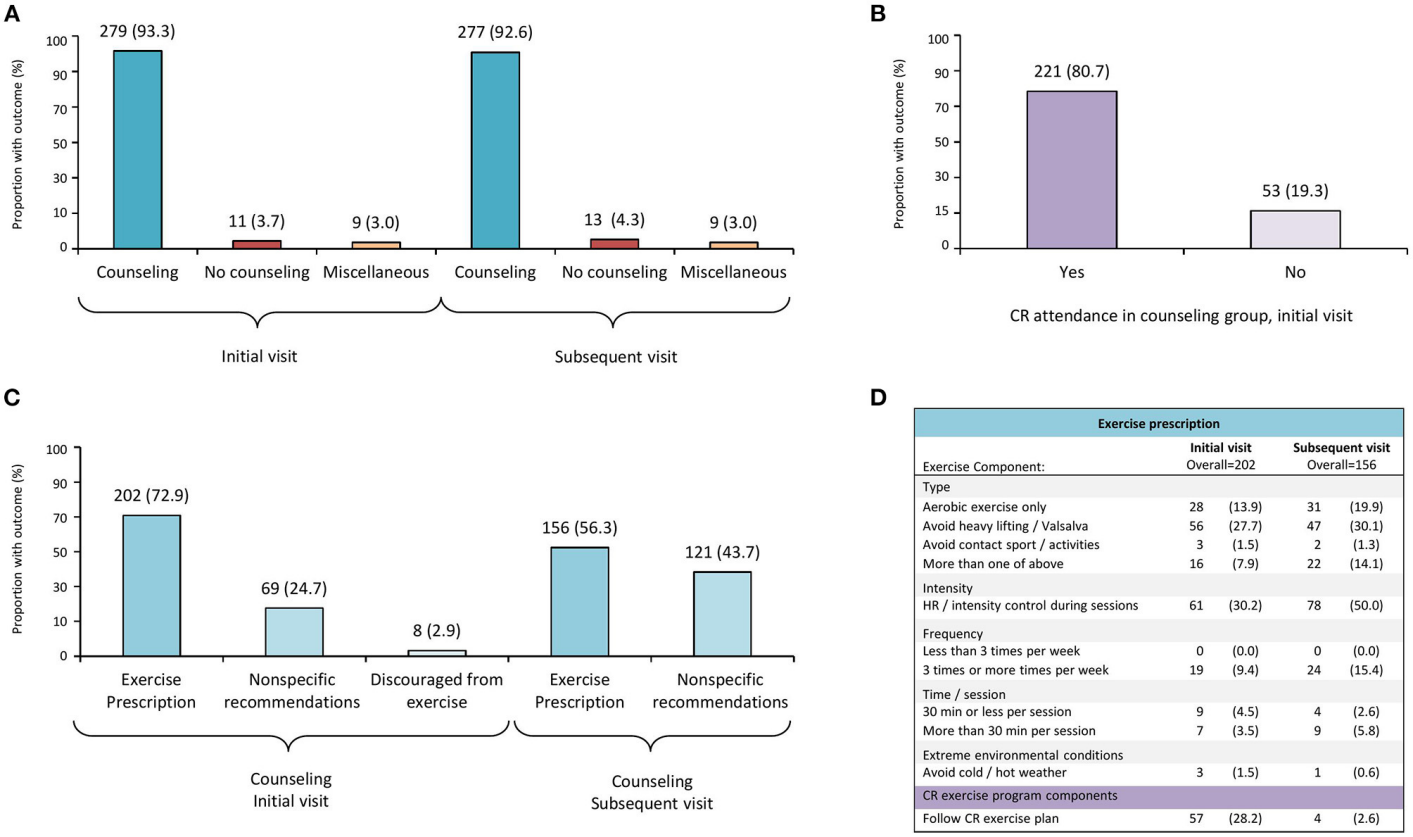
Physical activity counseling and exercise prescription patterns after SCAD. CR, cardiopulmonary rehabilitation; SCAD, spontaneous coronary artery dissection. Participants (299), who completed a follow-up survey after SCAD, provided details regarding physical activity counseling and exercise prescription received from their health care provider at first and subsequent medical encounter after SCAD. **(A)** Physical activity counseling at first and subsequent encounters after SCAD. **(B)** Proportion of participants who received physical activity counseling at initial visit after SCAD and attended cardiac rehabilitation (CR). **(C)** Subcategories of counseling received at first and subsequent encounters after SCAD. **(D)** Details of exercise prescription according to exercise components; CR category reflects participants who followed a CR exercise program and did not provide further details.

Figure 2A displays PA counseling at the initial and subsequent visits after SCAD. Most (*n* = 279, 93.3%) participants received PA counseling on their initial visit, namely, EXP (*n* = 202, 72.9%), non-specific recommendations (*n* = 69, 24.7%), or were discouraged from any exercise (*n* = 8, 2.9%) (Figure 2C).

During subsequent medical visits (Figure 2A), most (*n* = 277, 92.6%) participants were counseled, and 56.3% (*n* = 156) received EXP; nevertheless, 43.7% (*n* = 121) received non-specific recommendations (Figure 2C). No participants were discouraged from exercise at that time.

Figure 2D also shows EXP described by patients, according to exercise components (i.e., type, intensity, frequency, time/session, and extreme environmental exercise conditions) at initial and subsequent visits after SCAD.

Regarding exercise type, avoidance of heavy lifting and/or Valsalva maneuvers was the most common advice reported by participants (27.7 and 30.1% at initial and subsequent visit, respectively); <20% (*n* = 28 at initial visit; *n* = 31 at subsequent visit) were advised to perform only aerobic exercise, and avoidance of contact sports/activities was less likely reported at neither initial (*n* = 3, 1.5%) nor subsequent (*n* = 2, 1.3%) visits (Figure 2D).

Exercise intensity control or heart rate control during exercise sessions were more commonly reported among the subsequent visit (*n* = 78, 50.0%) recommendations (Figure 2D). In regard to exercise frequency and time/session components, fewer than 10% of patients described receiving recommendations at their initial visit, and only 15% (*n* = 24) of the participants described receiving recommendations about exercise frequency (≥3 sessions per week) at the subsequent visit. Less than 2% of the participants described receiving recommendations about avoiding extreme environmental conditions during exercise sessions on their initial or subsequent visit (i.e., avoidance of extreme cold/hot weather). Participants who reported they were told to follow a CR exercise program at their initial (*n* = 57, 28.2%) or subsequent visit (*n* = 4, 2.6%), but did not provide specific details regarding exercise components, are shown in Figure 2D.

## Discussion

To our knowledge, this study is the first to assess PA habits and EXP in the largest multinational registry of patients with confirmed SCAD to date. Insights from our study demonstrate the following: less than half of the participants were active prior to SCAD, and some of those were inclined to stop exercising after SCAD. Unusual PA was uncommon prior to SCAD. Most participants received some form of counseling PA after SCAD; however, specific advice regarding exercise components (i.e., type, intensity, frequency, time/session, and extreme environmental exercise conditions) was not consistently delivered at initial or subsequent visits after SCAD. Most participants have one or more traditional cardiovascular risk factor such as hypertension, dyslipidemia, diabetes, or elevated body weight. Recurrent symptoms of chest pain, chest discomfort, or shortness of breath following PA were not uncommon after SCAD, and survivors were concerned about recurrent SCAD events or sudden death in the future.

Patients with SCAD have been described as young, fit, active, mostly women, with few or unknown traditional risk factors for cardiovascular disease (6, 19, 23–25). We report in this study that most (70.1%) participants had at least one traditional risk factor for which exercise training during CR has shown to be beneficial (26). Prior to SCAD, 48.5% of the participants in our study performed aerobic exercise at least three times per week, and most (82.2%) of those performed exercise sessions ≥31 min; however, only 32.0% performed some strength-building exercises. The latest US monitoring data (27) showed that only 19% of women and 26% of men achieve sufficient activity defined by the 2018 Physical Activity Guidelines for Americans (28) as a combination of ≥150 min of moderate aerobic activities preferably spread through the week and 2 days of strength-building exercises per week. Our findings suggest that some SCAD patients achieve a reasonable amount of activity when compared with the adult US population; however, most of them may not be as active as assumed. This is particularly concerning since sparse exercise does not lead to optimal health (29), and active patients more often attend CR after the first SCAD, compared with less active patients (30).

Regarding PA counseling after SCAD, our study shows that most (93.3%) participants received PA advice at their first visit, and CR attendance among this group was substantial (Figure 2B), which may reflect either a motivated patient group and/or response bias. Among counseled patients in our study, specific advice delivered after SCAD about exercise components varied. At times, patients reported non-specific recommendations such as “listen or pay attention to your body,” “exercise as tolerated,” or “do what feels OK within reason,” especially on their subsequent (43.7%) medical encounter. Such recommendations may allow tailoring of physical effort according to patient's symptoms and to encourage them to learn to assess and adjust based on their perceived exertion. This can also be an alternative to constant monitoring of vital signs during exercise, which can be impractical, anxiety provoking, and frustrating. On the other hand, the disadvantage of such advice is that it may be unclear and potentially confusing for patients.

Previous reports have suggested extreme physical or emotional stress as possible precipitants for SCAD (6, 18). More often, strenuous physical stressors such as intense isometric exercise and weightlifting have been reported among men (6, 18). Most (68.3%) participants in our study did not report engaging unusual activities prior to SCAD. Fears of physical exertion triggering a recurrent dissection understandably may lead to advice against certain PA such as avoiding lifting or Valsalva maneuvers, not pursuing an activity beyond moderate aerobic exercise, or maintaining strict intensity control during PA (18). The mechanisms by which these stressors may contribute to SCAD are uncertain, and hypotheses have been based on experiences in patients with aortic diseases and individual SCAD case reports (31–33). Similarly, SCAD patients generally are advised against competitive or contact sports, exercising to the point of exhaustion, or performing exercise in extreme temperatures (hot yoga and cold weather) (4, 5, 34). These recommendations were rarely (1.5% or less) reported by our participants, which could be explained by the nature of our survey since we are relying on qualitative data generated through open-ended questions. Progress will require prospective studies to address this constraint.

Our study shows that lack of PA must be addressed in SCAD patients, and this could contribute to improved health and decrease their risk of future cardiometabolic diseases (28). SCAD patients are predominantly younger women who have received a heart disease diagnosis that places them in a high-risk population for psychological sequelae (35). Our group previously (36) reported significant prevalence of posttraumatic stress disorder, depression, and anxiety symptoms among SCAD survivors, and the severity of symptoms was worse among those who were younger at the time of SCAD. Likewise, our study shows that one-third of the patients report concerns regarding recurrence or sudden death after SCAD. This may or may not impact patient's decisions regarding PA practices after SCAD; however, close attention should be paid to previously active patients who have refrained from exercising after SCAD.

Moderate PA in all individuals with cardiovascular disease, after appropriate risk stratification, is recommended as a central component of therapy and is associated with reduced anxiety, hospital readmission, and cardiovascular and all-cause mortality (37). However, it is yet difficult to place these recommendations in SCAD patients since predicting which patients could have recurrent SCAD is not possible. Recently, the 2020 European Society of Cardiology guidelines on sports cardiology and exercise in patients with cardiovascular disease (37) recommend management of SCAD patients based on individual assessment. Resumption of leisure-time activities or competitive sports was suggested in patients under conservative management (i.e., without revascularization) who remain symptom-free, without evident inducible myocardial ischemia or with complete healing on computed tomography. However, exercise stress testing and coronary imaging are not standards of care prior to exercise resumption in SCAD patients (34). We acknowledge the inherent difficulties in formulating specific recommendations in this group of patients, and these recommendations denote progress in our understanding regarding SCAD management.

SCAD represents a challenge not only for health care teams but for patients who face unique issues concerning their disease due to little familiarity with the cause of their condition, uncertainty about treatment and management, doubt about the risk of recurrent events, and future decisions especially regarding lifestyle changes. Moreover, there is a substantial gap in evidence to guide clinicians regarding EXP after SCAD. To date, no SCAD-tailored physical activity guidelines have been created, and given the low prevalence of SCAD, randomized controlled trials to support evidence-based approaches have been difficult. Consequently, PA recommendations in SCAD are derived mostly from expert opinion, clinical experience, or CR evidence (9, 38). We hope that future larger-scale prospective studies help further our understanding of risk factors associated with SCAD recurrence and to fully comprehend patient's limitations regarding PA.

## Limitations

There are important limitations to consider in this study. First, this analysis is limited by selection and referral bias, and the registry's design prevented us from exploring multivariate associations. Second, most of the exercise data were self-reported; therefore, this study is limited by recall bias. Furthermore, qualitative data generated through open-ended questions were coded and categorized; this may be limited by errors in interpretation or analysis. Although the initial survey response rate was high, up to the time of this study, we were unable to obtain follow-up information from some participants, and only participants that had a complete set of data relevant to our aims were included. This could limit our findings and suggests there may be bias. Additionally, participants' questionnaires were collected at a single time point, which varied from the time of SCAD and may affect generalizability of the findings. However, the inclusion of patients from a variety of locations may help improve this generalizability. Despite all limitations, this study is the first study to describe PA habits and EXP in the largest series of patients with SCAD and serves to increase both patient and clinician's awareness of the physical activity and exercise habits in SCAD patients.

## Conclusion

Despite increased recognition of SCAD, knowledge gaps remain including optimal levels of PA and exercise following SCAD. Insights from our study suggest that only 48% of patients performed some aerobic exercise three or more times per week, and only 32.0% performed strength-building exercises, which suggest that most of them may not be as active as assumed. Furthermore, 70% of SCAD patients have one or more traditional cardiovascular risk factors. According to our findings, we suggest guiding patients based on individual assessment, taking into consideration baseline PA habits, treatment, and individual risk factors. Severely restricting activities or exercise sessions is potentially counterproductive and frustrating, and may contribute to the development of risk factors associated with a sedentary lifestyle.

Resumption of regular, moderate PA is likely beneficial in patients without recurrent symptoms or signs of ischemia and/or dissection. SCAD-tailored physical activity guidelines are needed for optimal exercise prescription without compromising patient safety.

## Data Availability Statement

The raw data supporting the conclusions of this article will be made available by the authors, without undue reservation.

## Author Contributions

AC-S, AB, JM-I, RG, PB, SH, and MT contributed to the conception or design of the work, drafted the manuscript, and critically revised the manuscript. RG, SH, and MT contributed to the acquisition and interpretation of data. AC-S, AB, and J-MI contributed to analysis and interpretation. All gave final approval and agree to be accountable for all aspects of work ensuring integrity and accuracy.

## Conflict of Interest

The authors declare that the research was conducted in the absence of any commercial or financial relationships that could be construed as a potential conflict of interest.

## References

[1] Tan NY Tweet MS. Spontaneous coronary artery dissection: etiology and recurrence. Expert Rev Cardiovasc Ther. (2019) 17:497–510. 10.1080/14779072.2019.1635011 31232618

[2] Tweet MS Gulati R Hayes SN. What clinicians should know about spontaneous coronary artery dissection. Mayo Clin Proc. (2015) 90:1125–30. 10.1016/j.mayocp.2015.05.010 26250728

[3] Nakashima T Noguchi T Haruta S Yamamoto Y Oshima S Nakao K. Prognostic impact of spontaneous coronary artery dissection in young female patients with acute myocardial infarction: a report from the angina pectoris-myocardial infarction multicenter investigators in Japan. Int J Cardiol. (2016) 207:341–8. 10.1016/j.ijcard.2016.01.188 26820364

[4] Adlam D Alfonso F Maas A Vrints C Writing C. European society of cardiology, acute cardiovascular care association, SCAD study group: a position paper on spontaneous coronary artery dissection. Eur Heart J. (2018) 39:3353–68. 10.1093/eurheartj/ehy080 29481627 PMC6148526

[5] Hayes SN Kim ESH Saw J Adlam D Arslanian-Engoren C Economy KE. Spontaneous coronary artery dissection: current state of the science: a scientific statement from the American Heart Association. Circulation. (2018) 137:e523–57. 10.1161/CIR.0000000000000564 29472380 PMC5957087

[6] Tweet MS Hayes SN Pitta SR Simari RD Lerman A Lennon RJ. Clinical features, management, and prognosis of spontaneous coronary artery dissection. Circulation. (2012) 126:579–88. 10.1161/CIRCULATIONAHA.112.105718 22800851

[7] Lavie CJ Milani RV. Cardiac rehabilitation and exercise training in secondary coronary heart disease prevention. Progr Cardiovascular Dis. (2011) 53:397–403. 10.1016/j.pcad.2011.02.008 21545925

[8] Dalal HM Doherty P Taylor RS. Cardiac rehabilitation. BMJ. (2015) 351:h5000. 10.1136/bmj.h5000 26419744 PMC4586722

[9] Silber TC Tweet MS Bowman MJ Hayes SN Squires RW. Cardiac rehabilitation after spontaneous coronary artery dissection. J Cardiopulm Rehabil Prev. (2015) 35:328–33. 10.1097/HCR.0000000000000111 25730096

[10] Thomas RJ Brewer LC. Strengthening the evidence for cardiac rehabilitation benefits. JAMA Cardiol. (2019) 4:1259–60. 10.1001/jamacardio.2019.4077 31642865 PMC7296560

[11] Williams MA Ades PA Hamm LF Keteyian SJ LaFontaine TP Roitman JL. Clinical evidence for a health benefit from cardiac rehabilitation: an update. Am Heart J. (2006) 152:835–41. 10.1016/j.ahj.2006.05.015 17070142

[12] Hammill BG Curtis LH Schulman KA Whellan DJ. Relationship between cardiac rehabilitation and long-term risks of death and myocardial infarction among elderly medicare beneficiaries. Circulation. (2010) 121:63–70. 10.1161/CIRCULATIONAHA.109.876383 20026778 PMC2829871

[13] Hayes SN. Spontaneous coronary artery dissection (SCAD): new insights into this not-so-rare condition. Tex Heart Inst J. (2014) 41:295–8. 10.14503/THIJ-14-4089 24955045 PMC4060337

[14] El-Sherief K Rashidian A Srikanth S. Spontaneous coronary artery dissection after intense weightlifting UCSF Fresno Department of Cardiology. Catheter Cardiovasc Interv. (2011) 78:223–7. 10.1002/ccd.22904 21413128

[15] Saw J Humphries K Aymong E Sedlak T Prakash R Starovoytov A. Spontaneous coronary artery dissection: clinical outcomes and risk of recurrence. J Am Coll Cardiol. (2017) 70:1148–58. 10.1016/j.jacc.2017.06.053 28838364

[16] Marijon E Fressonnet R Haggui A Mousseaux E Redheuil A. Spontaneous coronary dissection of the left main stem after intense physical activity–regression under conservative strategy. Int J Cardiol. (2008) 128:e16–8. 10.1016/j.ijcard.2007.04.157 17689717

[17] Waterbury TM Tweet MS Hayes SN Saw J. Early natural history of spontaneous coronary artery dissection. Circ Cardiovasc Interv. (2018) 11:e006772. 10.1161/CIRCINTERVENTIONS.118.006772 30354594

[18] Saw J Aymong E Sedlak T Buller CE Starovoytov A Ricci D. Spontaneous coronary artery dissection: association with predisposing arteriopathies and precipitating stressors and cardiovascular outcomes. Circ Cardiovasc Interv. (2014) 7:645–55. 10.1161/CIRCINTERVENTIONS.114.001760 25294399

[19] Mortensen KH Thuesen L Kristensen IB Christiansen EH. Spontaneous coronary artery dissection: a Western Denmark Heart Registry study. Catheter Cardiovasc Interv. (2009) 74:710–7. 10.1002/ccd.22115 19496145

[20] Vanzetto G Berger-Coz E Barone-Rochette G Chavanon O Bouvaist H Hacini R. Prevalence, therapeutic management and medium-term prognosis of spontaneous coronary artery dissection: results from a database of 11,605 patients. Eur J Cardio-Thoracic Surg. (2009) 35:250–4. 10.1016/j.ejcts.2008.10.023 19046896

[21] Tweet MS Gulati R Aase LA Hayes SN. Spontaneous coronary artery dissection: a disease-specific, social networking community-initiated study. Mayo Clin Proc. (2011) 86:845–50. 10.4065/mcp.2011.0312 21878595 PMC3257995

[22] Medicine ACoS. ACSM's Guidelines for Exercise Testing and Prescription, 10 Edn. Philadelphia, PA: Lippincott Williams & Wilkins (2017).

[23] Thompson EA Ferraris S Gress T Ferraris V. Gender differences and predictors of mortality in spontaneous coronary artery dissection: a review of reported cases. J Invasive Cardiol. (2005) 17:59–61. https://www.ncbi.nlm.nih.gov/pubmed/15640544 15640544

[24] Rashid HN Wong DT Wijesekera H Gutman SJ Shanmugam VB Gulati R. Incidence and characterisation of spontaneous coronary artery dissection as a cause of acute coronary syndrome–A single-centre Australian experience. Int J Cardiol. (2016) 202:336–8. 10.1016/j.ijcard.2015.09.072 26426273

[25] Faden MS Bottega N Benjamin A Brown RN. A nationwide evaluation of spontaneous coronary artery dissection in pregnancy and the puerperium. Heart. (2016) 102:1974–9. 10.1136/heartjnl-2016-309403 27411842

[26] Fihn SD Blankenship JC Alexander KP. 2014 ACC/AHA/AATS/PCNA/SCAI/STS focused update of the guideline for the diagnosis and management of patients with stable ischemic heart disease: a report of the American College of Cardiology/American Heart Association Task Force on Practice Guidelines, and the American Association for Thoracic Surgery, Preventive Cardiovascular Nurses Association, Society for Cardiovascular Angiography and Interventions, and Society of Thoracic Surgeons. J Thorac Cardiovasc Surg. (2015) 149:e5–23. 10.1016/j.jtcvs.2014.11.002 25827388

[27] Piercy KL Troiano RP. Physical activity guidelines for americans from the US Department of Health and Human Services. Circ Cardiovasc Qual Outcomes. (2018) 11:e005263. 10.1161/CIRCOUTCOMES.118.005263 30571339

[28] US Department of Health and Human Services. Physical Activity Guidelines for Americans. 2nd Edn. Washington, DC: US Dept of Health and Human Services (2018).

[29] Arnett DK Blumenthal RS Albert MA. 2019 ACC/AHA Guideline on the Primary Prevention of Cardiovascular Disease: A Report of the American College of Cardiology/American Heart Association Task Force on Clinical Practice Guidelines. Circulation. (2019) 140:e596–646. 10.1161/CIR.0000000000000725 30879355 PMC7734661

[30] Krittanawong C Tweet MS Hayes SE Bowman MJ Gulati R Squires RW. Usefulness of cardiac rehabilitation after spontaneous coronary artery dissection. Am J Cardiol. (2016) 117:1604–9. 10.1016/j.amjcard.2016.02.034 27055757

[31] Erbel R Aboyans V Boileau C Bossone E Bartolomeo RD Eggebrecht H. 2014 ESC Guidelines on the diagnosis and treatment of aortic diseases: document covering acute and chronic aortic diseases of the thoracic and abdominal aorta of the adult. The Task Force for the Diagnosis and Treatment of Aortic Diseases of the European Society of Cardiology (ESC). Eur Heart J. (2014) 35:2873–926. 10.1093/eurheartj/ehu281 25173340

[32] Yiangou K Papadopoulos K Azina C. Heavy lifting causing spontaneous coronary artery dissection with anterior myocardial infarction in a 54-year-old woman. Tex Heart Inst J. (2016) 43:189–91. 10.14503/THIJ-15-5097 27127443 PMC4845561

[33] Ellis CJ Haywood GA Monro JL. Spontaneous coronary-artery dissection in a young woman resulting from an intense gymnasium work-out. Int J Cardiol. (1994) 47:193–4. 10.1016/0167-5273(94)90191-0 7721492

[34] Hayes SN Tweet MS Adlam D Kim ESH Gulati R Price JE. Spontaneous coronary artery dissection: JACC state-of-the-art review. J Am Coll Cardiol. (2020) 76:961–84. 10.1016/j.jacc.2020.05.084 32819471

[35] Tweet MS Kok SN Hayes SN. Spontaneous coronary artery dissection in women: what is known and what is yet to be understood. Clin Cardiol. (2018) 41:203–10. 10.1002/clc.22909 29493808 PMC5953427

[36] Johnson AK Hayes SN Sawchuk C Johnson MP Best PJ Rajiv Gulati. Analysis of posttraumatic stress disorder, depression, anxiety, and resiliency within the unique population of spontaneous coronary artery dissection survivors. J Am Heart Assoc. (2020) 9:e014372. 10.1161/JAHA.119.014372 32342736 PMC7428589

[37] Pelliccia A Sharma S Gati S Bäck M Börjesson M Caselli S. 2020 ESC Guidelines on sports cardiology and exercise in patients with cardiovascular disease. Eur Heart J. (2020) 42:17–96. 10.1093/eurheartj/ehaa605 32860412

[38] Chou AY Prakash R Rajala J Birnie T Isserow S Taylor CM. The first dedicated cardiac rehabilitation program for patients with spontaneous coronary artery dissection: description and initial results. Can J Cardiol. (2016) 32:554–60. 10.1016/j.cjca.2016.01.009 26923234

