# Supplementary material for: Physical Activity and Exercise Patterns After Spontaneous Coronary Artery Dissection: Insights From a Large Multinational Registry
Source: Front Cardiovasc Med. 2021 Jun 15;8:642739. doi: 10.3389/fcvm.2021.642739 (PMC8240509; doi:10.3389/fcvm.2021.642739)
Supplement: Supplementary file 1 [file Data_Sheet_1.docx]

Supplementary Material

## Supplemental Figures


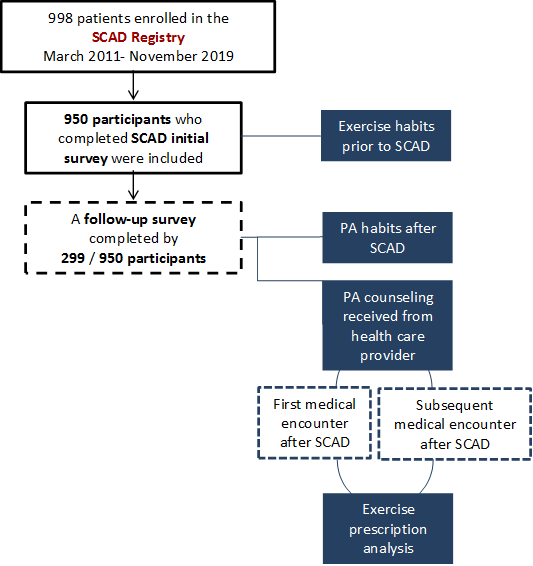


**Supplemental Figure 1:** CONSORT flow diagram.

PA, physical activity; SCAD, spontaneous coronary artery dissection.

| **Exercise Prior to SCAD** | |
| --- | --- |
| 8. In an **average week the month prior to your SCAD event**,  a. How many times did you exercise aerobically (physical activity lasting at least 20 minutes, during which you breathe more heavily and your heart beats faster)?  If yes, how long was your typical exercise session?  b. In the month prior to your SCAD event, about how many hours per week did you do non-aerobic exercise, such as bowling, softball, golf, walking, or yard work?  c. Did you regularly do strength building exercises with resistance or weight lifting equipment?  d. Did you engage in any unusual physical activity prior to your SCAD event? | ○ None  ○ Less than once per week  ○ 1 or 2 times per week  ○ 3 or more times per week  ○ Not Sure  ○ Less than 20 minutes  ○ 21-30 minutes  ○ 31-60 minutes  ○ More than 60 minutes  ○ Not Sure  ○ None  ○ Less than 1 hour  ○ 1 hour  ○ 2 hours  ○ 3 hours  ○ 4 hours or more  ○ Not Sure  ○ Yes ○ No ○ Not sure  ○Yes, please describe:    ○No ○ Not Sure |

**Supplemental Figure 2:** Initial SCAD Survey.

Min, minutes; SCAD, spontaneous coronary artery dissection


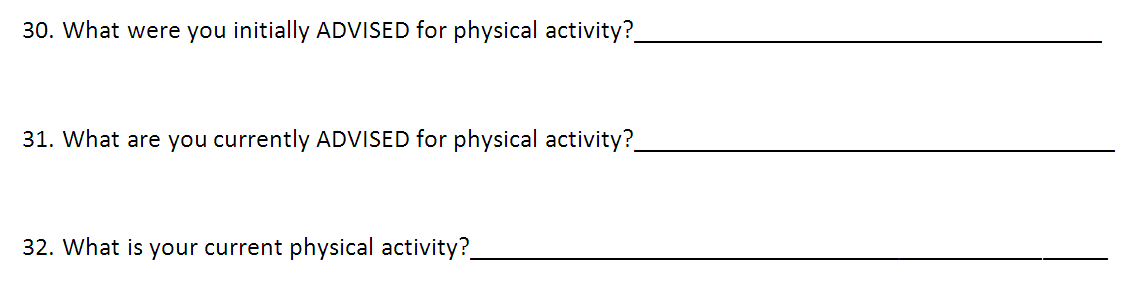

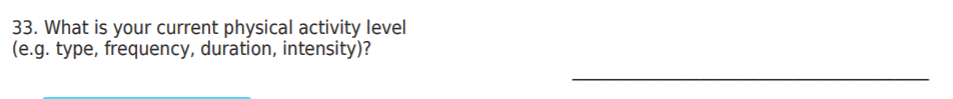


**Supplemental Figure 3:** SCAD follow-up survey.

Min, minutes; SCAD, spontaneous coronary artery dissection


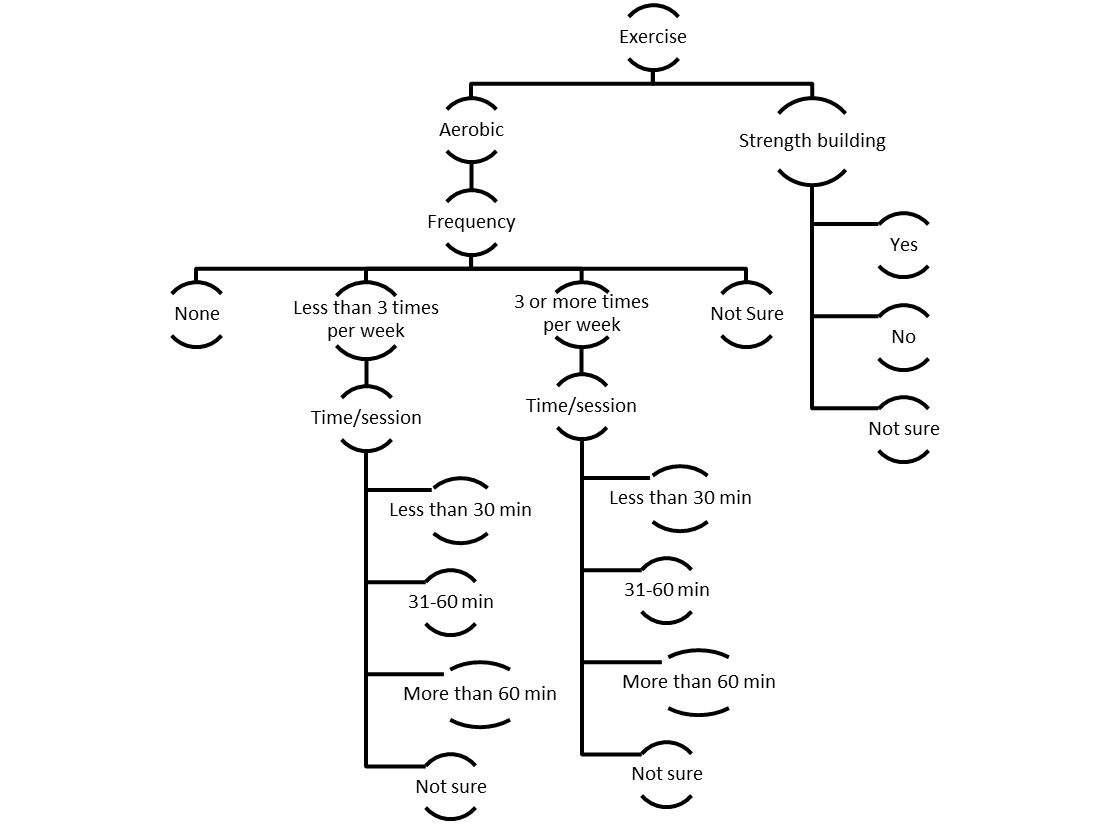


**Supplemental Figure 4:** Categorization and grouping of quantitative data from SCAD first survey.

Min, minutes; SCAD, spontaneous coronary artery dissection


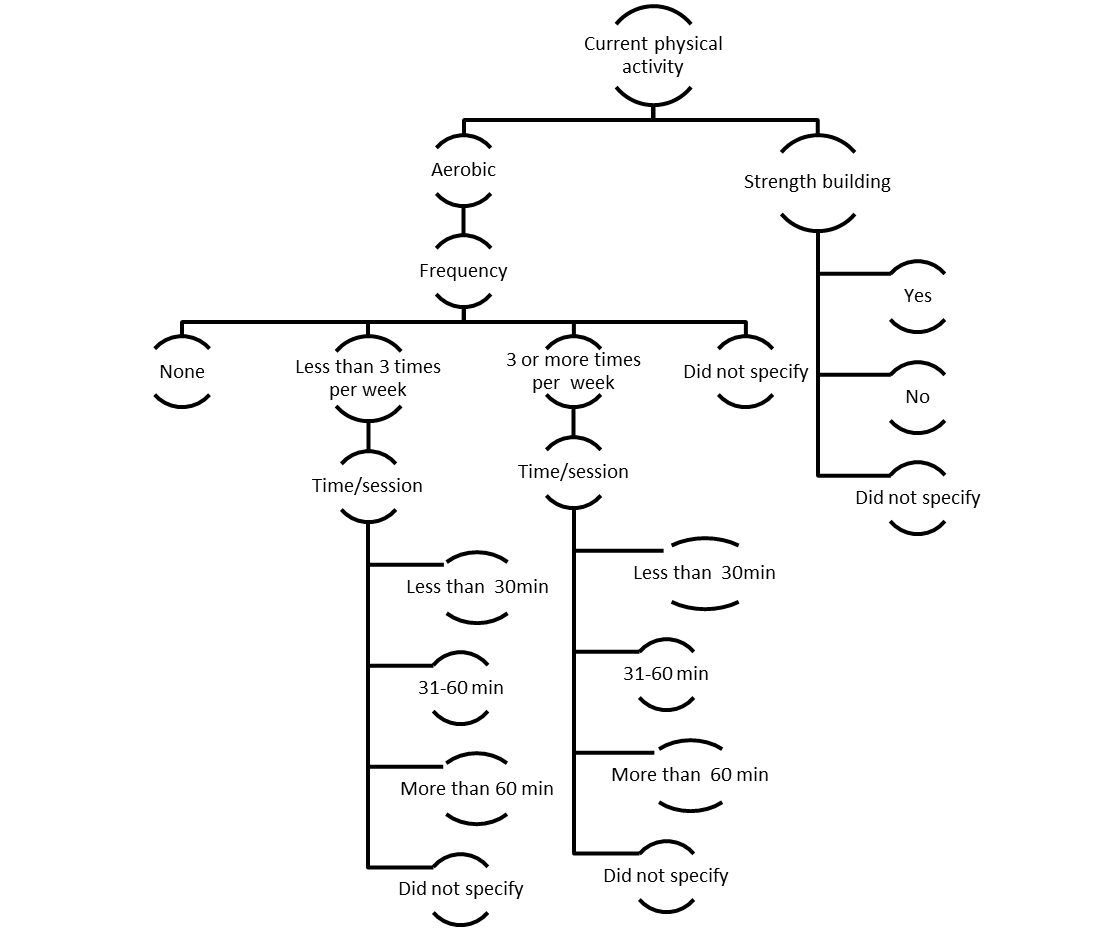


**Supplemental Figure 5:** Theoretical framework for open coding during qualitative analysis of SCAD Follow up survey.

Min, minutes; SCAD, spontaneous coronary artery dissection.


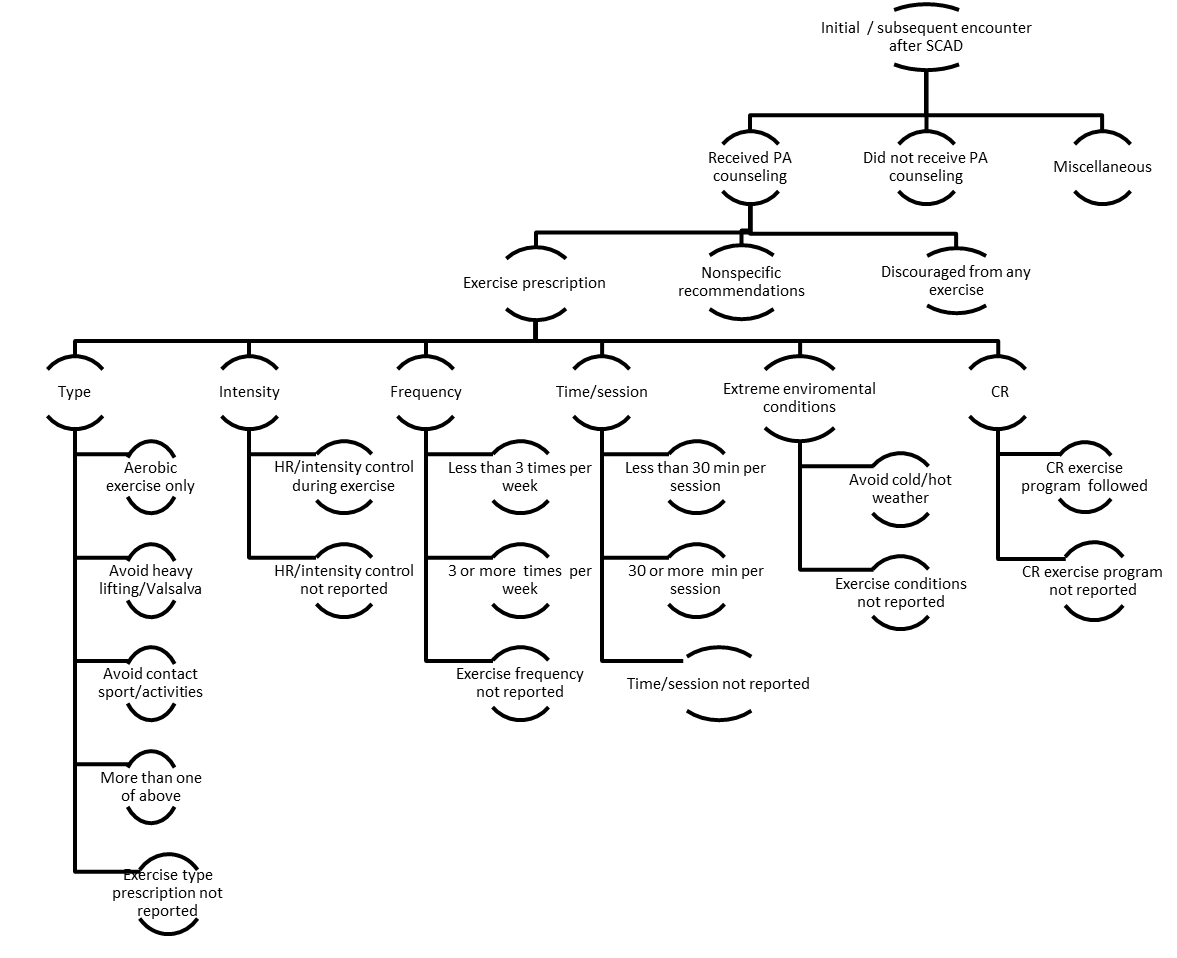


**Supplemental Figure 6:** Theoretical framework for open coding during qualitative analysis of first and subsequent encounters data, from SCAD follow up survey.

Min, minutes; PA, physical activity; SCAD, spontaneous coronary artery dissection.


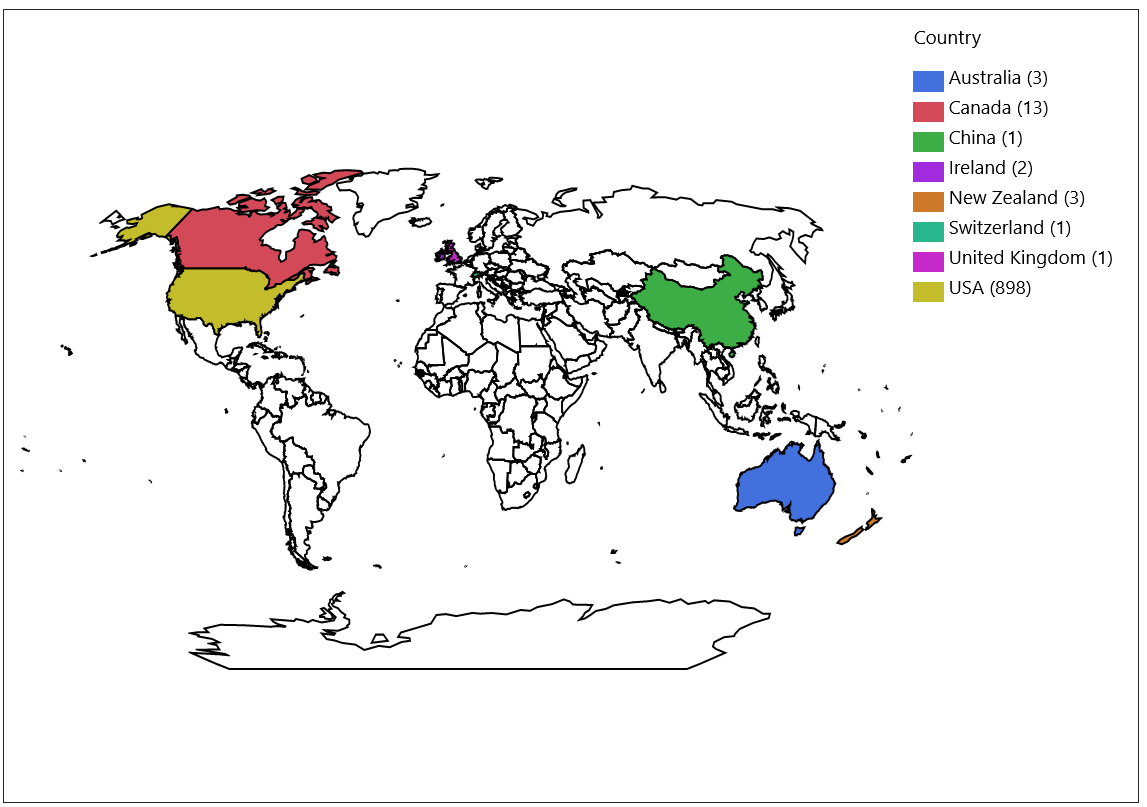


**Supplemental Figure 7:** Geographic distribution of patients with SCAD (n=922). Please note, 28 participants did not disclose this information.

SCAD, spontaneous coronary artery dissection.
